# Omega-3 Fatty Acids and Traumatic Injury in the Adult and Immature Brain

**DOI:** 10.3390/nu16234175

**Published:** 2024-11-30

**Authors:** Ester Valero-Hernandez, Jordi L. Tremoleda, Adina T. Michael-Titus

**Affiliations:** Centre for Neuroscience, Surgery and Trauma, Blizard Institute, Barts and The London School of Medicine and Dentistry, Queen Mary University of London, London E1 2AT, UK; e.valerohernandez@qmul.ac.uk (E.V.-H.); j.lopez-tremoleda@qmul.ac.uk (J.L.T.)

**Keywords:** traumatic brain injury, omega-3 polyunsaturated fatty acids, docosahexaenoic acid, eicosapentaenoic acid, neuroprotection

## Abstract

**Background/Objectives:** Traumatic brain injury (TBI) can lead to substantial disability and health loss. Despite its importance and impact worldwide, no treatment options are currently available to help protect or preserve brain structure and function following injury. In this review, we discuss the potential benefits of using omega-3 polyunsaturated fatty acids (O3 PUFAs) as therapeutic agents in the context of TBI in the paediatric and adult populations. **Methods:** Preclinical and clinical research reports investigating the effects of O3 PUFA-based interventions on the consequences of TBI were retrieved and reviewed, and the evidence presented and discussed. **Results:** A range of animal models of TBI, types of injury, and O3 PUFA dosing regimens and administration protocols have been used in different strategies to investigate the effects of O3 PUFAs in TBI. Most evidence comes from preclinical studies, with limited clinical data available thus far. Overall, research indicates that high O3 PUFA levels help lessen the harmful effects of TBI by reducing tissue damage and cell loss, decreasing associated neuroinflammation and the immune response, which in turn moderates the severity of the associated neurological dysfunction. **Conclusions:** Data from the studies reviewed here indicate that O3 PUFAs could substantially alleviate the impact of traumatic injuries in the central nervous system, protect structure and help restore function in both the immature and adult brains.

## 1. Traumatic Brain Injury—Epidemiological Data and Impact

Traumatic brain injury (TBI) is a neurological condition triggered by impact of an external force on the brain through direct contact or shock waves—such as the blast waves following an explosion. Around 69 million people worldwide sustain a TBI yearly, and it remains a global health challenge [1,2]. It occurs in a civilian or military context, the importance of the latter being particularly apposite considering the numerous active zones of conflict worldwide. Death and disability rates are higher than in any other forms of traumatic injury, having a major impact in those aged under 40, but the epidemiology of TBI is changing and reflects the ageing trend of the world population, especially in high-income countries [2]. The increase in the number of older people with TBI and comorbid medical conditions amplifies the public health impact.

The leading causes of TBI are road accidents and falls [3], with the latter being more common in older adults and often linked to sarcopenia, osteoporosis, frailty and multimorbidity, which can hinder recovery [4,5]. The socioeconomic impact of TBI is significant, as TBI can lead to long-term complications such as secondary epilepsy, chronic headaches, anxiety, depression, cognitive dysfunction, and endocrine problems [6,7,8,9,10]. An important consequence of TBI is the increased risk of developing neurodegenerative diseases, such as Alzheimer’s disease (AD), which is a major contributor to the global burden of disease [11], Parkinson’s disease (PD) [12], or amyotrophic lateral sclerosis (ALS) [13,14], which altogether indicates that TBI induces long-lasting changes in the brain [15]. Injury induced through repeated head concussion (a form of mild TBI), such as that experienced in contact sports, e.g. American football, boxing, or rugby, is associated with an increased risk of developing chronic traumatic encephalopathy (CTE), a neuropathologically distinct form of neurodegeneration [16,17,18].

TBI is also a leading cause of death and disability in children and adolescents [19,20], but this is understudied compared to adult TBI [21]. Within the paediatric population, there are two peaks in the incidence of TBI: in children between 0 and 4 years of age, and in adolescents and young adults between the ages of 15 and 24 years [22]. Causes include falls, motor vehicle collisions, injuries related to sports, and also injuries linked to non-accidental trauma and child abuse [23,24]. Similar to adult TBI, the most common type of injury in the paediatric population is mild TBI, and although the majority of cases will recover rapidly, more than 20% of adult or paediatric patients endure persistent symptoms beyond the first year following injury [25,26,27,28]. Concussions, a form of mild TBI, are a common occurrence in the context of school and university sports [29], and adolescent athletes that are prematurely cleared for return-to-play are at increased risk of suffering the long-term effects of repeated concussions. A “second impact syndrome” may occur when athletes sustain a second head injury before the initial concussion has completely resolved—this can lead to rapid diffuse cerebral swelling, brain herniation, and death [30].

Despite TBI being a global public health concern, current treatment only focuses on symptom management, and no neuroprotective agents with an aim to protect or preserve brain structure and function after injury have been approved for use in the clinic [31]. This review discusses the impact of interventions using omega-3 polyunsaturated fatty acids (O3 PUFAs) as neuroprotective agents for the alleviation of the effects of TBI and summarises the evidence from preclinical and clinical studies carried out so far.

## 2. Present Management of TBI

TBI is an extremely heterogeneous condition that encompasses different types of injuries, such as contusions, haematomas, subarachnoid haemorrhage, and diffuse axonal injury, and different severity levels. To acutely assess the severity of the injury, a scoring system known as the Glasgow Coma Scale (GCS) is used, which measures three aspects of responsiveness (eye-opening, verbal and motor responses) and classifies severity into “mild”, “moderate”, and “severe”. A GCS for children is available, with appropriate modifications that reflect developmental differences. The 8-point Glasgow Outcome Scale extended (GOS-E) is a commonly used score that helps assess the outcome of TBI, where a score of 1 is “dead” and a score of 8 is “full recovery”. While the GCS and GOS-E help assess severity and outcome, there is an increasing recognition that these scales are too simplistic as categorisation systems and fail to capture the heterogeneity of TBI [32,33]. To address this, there is effort to develop better approaches for prognosis, such as the International Mission for Prognosis and Clinical Trial Design (IMPACT) score and the Corticosteroid Randomisation After Significant Head Injury (CRASH) score [34].

Since the injured brain is highly vulnerable to systemic insults such as hypoxia, hypotension, and coagulopathy, the current management of TBI in the acute phase, i.e. minutes to hours after injury, by pre-hospital and trauma teams puts emphasis on the stabilization of physiological parameters, such as monitoring and controlling intracranial pressure and blood pressure, correcting injury-related coagulopathy, evacuating mass lesions, and ensuring adequate ventilation [35]. Improvements in trauma care systems and supportive critical care have resulted in a dramatic reduction in mortality rates post-TBI in hospitals with appropriate specialist support, from 80% in the 1940s to 20% at present [36]. Following the acute stage, the rehabilitation offered to patients (often focused on physiotherapy, speech and occupational therapy, and sometimes psychological therapy) is variable between centres but aims to support patients towards long-term recovery [37].

## 3. Neuropathological Mechanisms of TBI

A traumatic event where a direct or indirect force affects the brain can trigger a host of pathophysiological processes that unfold over days and weeks after injury, in two distinct stages [38,39,40]. The immediate impact of the injury, described as the “primary injury,” is characterised by significant tissue deformation and tear, meningeal damage, necrotic cell death, axonal shearing, and damage to blood vessels, leading to significant impairment of the vascular supply and disrupted integrity of the blood–brain barrier (BBB). Immediately after injury, damaged cells release intracellular components into the extracellular space—these are known as damage-associated molecular patterns (DAMPs) and are recognised by cells of the innate immune system. Examples of DAMPs include ATP, heat shock proteins (HSPs), reactive oxygen species (ROS), and high mobility group box protein 1 (HMGB1) [41,42]. There is also activation of matrix metalloproteinases (MMPs), such as MMP2 and MMP9, which exacerbate the degradation of the tissue extracellular matrix and the damage to the BBB [43,44].

These first consequences of injury are subsequently exacerbated by processes that are described as the “secondary injury”, which include excessive glutamate activity (excitotoxicity), apoptotic death, metabolic changes (initial increase in glucose uptake followed by cerebral glucose hypometabolism) [45], activation of resident glial cells (microglia and astrocytes), cytokine and chemokine release, mitochondrial dysfunction, oxidative stress, and activation of a complex systemic immune response, including brain ingress of peripheral immune cells and complement activation [46,47]. In the CNS, the major innate immune cell type is the microglia [48], which respond to DAMPs by transforming to an “activated” state, covering a spectrum that historically has been described as ranging from the M1 state, or “classically activated”, to the M2 state, or “alternatively activated”. The M1 phenotype is viewed as proinflammatory and is characterised by the expression of surface markers CD32 and CD86 and the release of pro-inflammatory cytokines (such as tumour necrosis factor-α (TNF-α), interleukin (IL)-1β, and ROS, whereas M2 is an anti-inflammatory phenotype characterised by the expression of surface markers CD206 and arginase 1, and the production of anti-inflammatory cytokines (such as IL-4, IL-10, and IL-13). In the context of TBI, M1 microglia are associated with exacerbation of the injury, whereas M2 microglia are associated with repair processes; however, the M1/M2 dichotomy is widely acknowledged to be rather a simplification that does not reflect the existence of intermediate, evolving complex phenotypes [49,50]. Astrocytes are the other key players in the local neuroinflammatory response following injury [51], and they also respond to DAMPs by undergoing molecular, structural, and functional changes that characterise the hypertrophic reactive astrogliosis that occurs post-injury [52]. The TBI-induced increase in DAMPs causes an acute rise in cytoplasmic calcium within the reactive astrocyte networks surrounding the injury site, and this precedes the polarization of astrocyte processes towards the injury site. Glial fibrillary acidic protein (GFAP) expression, a major component of glial intermediate filaments, increases markedly in astrocytes following their activation [53]. A glial scar formed by reactive astrocytes is gradually established around the injury site, which initially has a protective role and limits the spread of the injury but later on hinders neural repair [54]. In microglia and astroglia, some of the direct targets DAMPs act on are the NOD-, LRR-, and pyrin domain-containing protein 3 (NLRP3) inflammasome [55] and the nuclear factor kappa B (NF-κB) transcription factor [56]. Imaging studies and analyses of post-mortem brain tissue of people that sustained a TBI showed persisting neuroinflammation decades after the initial injury, which may be linked to the white matter injury, oligodendrocyte loss, demyelination, and degeneration that occurs post-TBI [57,58,59].

Apart from the local immediate immune response in the brain parenchyma, TBI also triggers a systemic response [60]. Blood-borne leukocytes migrate to the damaged tissue, with the first responders being neutrophils that rapidly migrate to clear cellular debris, followed by macrophages, NK and dendritic cells, and T and B cells [61,62]. This complex response can ultimately have both injury-amplifying effects and pro-repair potential [60,63]. As part of the inflammatory response, an array of cytokines and chemokines are secreted following traumatic injury [64]. Within the first hours post-injury in the adult brain, there is an increase in cytokines such as TNF-α, IL-1β, and IL-6, chemokines such as growth-regulated oncogene (GRO) (known as keratinocyte chemoattractant (KC) in mice and cytokine-induced neutrophil chemoattractant (CINC) in rats) and monocyte chemoattractant protein-1 (MCP-1) (also known as C-C motif ligand 2 (CCL-2)), and alarmins such as galectin-3 [65,66]. Increases in cytokine levels (such as IL-6 and IL-12) have also been reported in paediatric TBI within the first few hours to days post-injury [67], with higher IL-6 values found to be correlated with poorer GCS scores.

TBI is also associated with oxidative stress, which reflects an imbalance between the production of oxidation-derived free radicals (such as superoxide anions, hydroxide radicals, and hydrogen peroxide) and the scavenging of these species through cellular antioxidant systems, such as the enzymes superoxide dismutase (SOD), glutathione peroxidase and catalase, and compounds like glutathione [68]. Finally, the evolution of the injury is often complicated by additional adverse events, such as a reduction in systemic blood pressure, the development of intracranial hypertension, brain hypoxia, and hypoperfusion, which can exacerbate ischaemia in the grey and white matter.

There is some evidence that the events post-injury may differ in the immature brain, i.e. in children and adolescents. Children have a thinner and more pliable skull, a relatively large head, and weak neck musculature, which make them more susceptible to shear and rotational acceleration forces [69]. Injury in the immature brain differs significantly from injury of a mature brain due to numerous developmental, pre-injury, and injury-related factors that can affect recovery while the brain continues to develop. Studies on paediatric injury over a period of 6 to 24 years post-injury showed that people who had a TBI early in life, within the period spanning from the toddler stage to the first years of adolescence, were affected by impaired intellectual and academic abilities [70], attentional deficits [71], and deficits in emotional perception and socio-cognitive function [72,73], and were at higher risk of early onset of a psychiatric disorder [74]. Adolescence is intrinsically a time characterized by more impulsivity and risk-taking behaviour, and post-injury there is a higher propensity to abuse alcohol and use drugs [75]. There is also evidence that in paediatric TBI there are differences in the cellular and molecular pathophysiology of TBI, such as in the inflammatory response (for instance, in paediatric TBI there appears to be a decreased neutrophil infiltration response [76] but a more persistent presence of leukocytes in the brain [75]), as well as other mechanisms, such as increased apoptotic death, shorter hypometabolic phase, more intensive oxidative stress (linked to a reduced antioxidant reserve in severe TBI [77]), and more hemodynamic instability [78].

Overall, TBI-triggered processes lead to significant tissue loss and brain dysconnectivity. Cognitive domains such as memory, processing speed, and executive functions, all commonly impaired after injury, depend on the coordinated activity of widely distributed networks [79,80]. Coherence in these networks depends on the integrity of long white matter tracts; therefore, large-scale tract disruption is a critical determinant of the cognitive impairment that occurs in both adult and paediatric cases of TBI [81,82,83].

The use of various plasma biomarkers with diagnostic and/or prognostic value in the context of TBI has garnered attention over the years; as such, systemic biomarkers reflect damage to grey and white matter and the extent of injury of neuronal and non-neuronal cells. These include GFAP, S100B, myelin basic protein (MBP), neuron-specific enolase (NSE), neurofilament L (NFL), and ubiquitin carboxy-terminal hydrolase L1 (UCH-L1) [84]. GFAP, UCH-L1, and S100B have been shown to be of clinical benefit and have been incorporated into clinical guidelines to help decide if a CT scan is required [85]. Plasma TBI biomarkers also include cytokines such as IL-6, IL-1β, and IL-8, which reflect the inflammatory response and its evolution from the acute into the subacute phase [86]. In a recent study focused on the inflammatory response within 24 h post-TBI, a distinct plasma signature involving IL-15 showed significant potential for severity differentiation and prognosis [87]. Although useful, there are some limitations of these biomarkers, including their non-specificity for TBI (i.e. extracerebral sources), and possible interpersonal variability due to sex differences, the nature of the injury, or uneven diffusion across the BBB. Continued efforts are put into the identification of new biomarkers, e.g. exosomes and micro RNAs (miRNA) [88]. In addition to plasma analysis, neuroinflammation could be monitored through imaging, and as shown by Ramlackhansingh et al., an inflammatory response can be detected for years after injury [57].

The complex cascade of mechanisms unfolding after TBI offers multiple targets for neuroprotection of the adult and immature brain, but the field of neuroprotection in TBI has somewhat lost momentum following failures in the clinical translation of experimental data [31,36,89,90].

## 4. Omega-3 and Omega-6 Fatty Acids and Derivatives

The brain is one of the richest organs in lipid content in the body, and its lipids have great structural diversity [91]. Fatty acids (FAs) are hydrophobic compounds that consist of a hydrocarbon chain of varying length and a terminal carboxylic acid group (COOH), and they can be part of more complex lipids (e.g. phospholipids, triacylglycerol or cholesteryl esters), or be “free” (i.e. non-esterified) FAs (FFAs) [92]. Depending on the presence or absence of double bonds within their carbon chain, FAs can be classified into unsaturated or saturated FAs, respectively. Polyunsaturated FAs (PUFAs) contain two or more double bonds (the bonds are in cis, separated by a methylene group), and according to the position of the first double bond in regard to the methyl carbon, they can be further classified into two families: omega-3 (O3) and omega-6 (O6) PUFAs. FFAs serve as important energy sources for many tissues in the body but also function as signalling molecules that regulate various cellular processes and physiological functions through specific receptors [93,94].

The biosynthetic precursor of O3 PUFAs is the plant-derived essential FA α-linolenic acid (ALA), which has an 18-carbon chain and three double bonds (18:3n-3). A succession of elongation and desaturation reactions involving desaturase and elongase enzymes, and ultimately its β-oxidation, lead to the successive transformation of ALA into longer chain O3 PUFAs: stearidonic acid (18:4n-3), eicosatetraenoic acid (20:4n-3), eicosapentaenoic acid (EPA; 20:5n-3), docosapentaenoic acid (DPA; 22:6n-3), and docosahexaenoic acid (DHA: 22:5n-3) [95,96,97]. Similarly, linoleic acid (LA) serves as a precursor for the synthesis of the long-chain O6 PUFA arachidonic acid (AA; 20:4n-6). ALA and LA are regarded as essential fatty acids because they are synthesized in plants and need to be obtained from the diet [98]. However, the conversion of ALA into long-chain PUFAs is not very efficient; for instance, conversion of ALA into DHA is <1% in men and ~2% in women (although it increases up to 9% in pregnancy) [99,100,101]. For this reason, direct dietary intake of long-chain O3 PUFAs, instead of their precursor only, plays an important role in ensuring optimum levels are reached.

Numerous studies have explored the importance of DHA and EPA in brain health [102], but much less is known about the role of DPA [96]. Local synthesis of DHA, EPA, and DPA is low in the brain, so brain levels are largely maintained by their uptake from the plasma [103]. The major facilitator superfamily domain containing 2a (Mfsd2a) protein was identified as the main transporter of DHA across the BBB and into the brain, which selectively transports DHA in the form of lysophosphatidylcholine (LPC)-DHA, and not as free DHA [104]. This specific role of LPC-DHA had been previously reported by Thies and collaborators [105]. Importantly, DHA is found to be enriched in the brain and in much higher quantities than any other O3 PUFA [106], and low DHA brain levels have been associated with impaired brain function and memory, as well as neurodegenerative and neuropsychiatric conditions such as AD, PD, schizophrenia, and depression [107]. It is important to note that during human evolution the relative ratio of dietary intake of O6:O3 PUFAs has dramatically changed, increasing from around 4:1 in ancestral hunter–gatherer communities to 20:1 today [108], and these alterations in the O6:O3 ratio have been proposed to be associated with an increased risk for a variety of chronic diseases [109]. It is possible to have an indication of the dietary intake of O3 PUFAs, and in general the O3 status, by measuring the Omega-3 Index (O3I), which reflects the percentage of EPA and DHA relative to total fatty acids in erythrocyte membranes [110]. The optimal O3I range is 8–12%, but many people, especially in countries with Western dietary habits, have an index below 8% [111]. Evidence indicates that the O3I fulfils many of the requirements to be assessed as a risk factor for cardiovascular disease [110,112], and there is also evidence that a high O3 status is positively linked to higher executive function and cognitive flexibility in adults [113] and higher cognition in children [114].

Apart from their intrinsic effects, another important aspect of the life cycle of O3 and O6 PUFAs is their oxygenation and conversion into a large range of bioactive mediators, designated with the generic term of “oxylipins” [115,116]. The two main classes of oxylipins are eicosanoids, derived from C20 PUFAs (such as AA and EPA), and docosanoids, derived from C22 PUFAs (such as DHA). Membrane-derived phospholipid PUFAs, released by the action of phospholipase A2 (PLA2), are transformed into oxylipins via enzymatic reactions, involving cyclooxygenase (COX), lipoxygenase (LOX), or cytochrome P450 enzymes; mixed-function oxidase reactions; or non-enzymatically, via free radical-catalysed reactions [117,118]. Specific markers of oxidative stress post-TBI include PUFA-derived compounds produced non-enzymatically through peroxidation, such as 4-hydroxynonenal (4-HNE), derived from AA, and 4-hydroxyhexenal (4-HHE), derived from DHA [119,120]; these bioactive molecules can form adducts with DNA, proteins, and lipids. Oxylipins are a large and diverse family, including prostaglandins, leukotrienes, thromboxanes, mono-, di-, and tri-hydroxy fatty acids, epoxy fatty acids, eoxins, lipoxins, hepoxilins, maresins, protectins (also called neuroprotectins in the brain), and resolvins. The diversity of precursor FA and/or the oxylipin generation pathway followed determines their specific distinct functions [115]. Oxylipins have important roles in modulating immune function and inflammation in physiological conditions, and have effects on the vascular, immune, cardiac, and renal systems [115]. O3 PUFA-derived oxylipins have a major role in the resolution of inflammation, preventing excess inflammation and uncontrolled tissue damage [116,121,122]. In mice, dietary supplementation with O3 PUFAs leads to a pro-resolving brain oxylipin profile [123], and a shift in serum pro-resolving oxylipins has been shown in healthy volunteers after several weeks of supplementation with DHA and EPA [124].

## 5. Dynamics of PUFAs and Oxylipins Following Neurotrauma

TBI leads to a complex array of changes in lipids, both in the injured tissue and at the periphery [125]. Immediately following tissue injury and the release of DAMPs, which include AA-derived prostaglandins and leukotrienes, an inflammatory cascade is initiated. Later on, oxylipin metabolism switches to the production of specialized pro-resolving mediators (SPMs), which include lipoxins, resolvins, protectins, and maresins, which have important roles in inflammation resolution by limiting further neutrophil infiltration to the injury site and also stimulating the clearance of apoptotic neutrophils [122].

A study in a TBI model of mild fluid percussion injury (mFPI) in cats reported increased brain levels of prostaglandin (PG) E2 (PGE2), PGF2α, and 6-keto-PGF1α, up to 158% of control levels in the first hour post-injury [126], and increases in PGE2 and thromboxane B2 were observed 5 min after injury in a rat FPI model [127]. In a later study, it was found that a weight drop (WD) TBI in rats led to increased PLA2 enzymatic activity by 75% 15 min after trauma, and then 245% 24 h after injury, with consequent production of PGE2 [128]. Increased levels of brain FFAs were observed in the controlled cortical injury (CCI) rat model of TBI as early as 30 min after injury, characterised by a preferential hydrolysis of 20:4-phospholipids first, releasing AA, followed by 22:6-phospholipid hydrolysis one day after the injury, releasing DHA [129,130]. Mass spectrometry imaging analysis showed a rapid decrease in DHA-containing phospholipids near the injury site in the first 3 days after rat CCI [131]. In rat FPI, Guo and colleagues showed that DHA-containing brain phospholipids at the injury site increased in the subacute phase, i.e. 5–7 days post-injury, suggesting a potential link with neurorepair mechanisms [132]. In contrast, in a study examining changes 3 months after CCI in mice, a persistent decrease was reported in DHA-containing phospholipid species in the hippocampus, cortex, and plasma [133].

In humans, increases in FFA levels following TBI have been detected in the cerebrospinal fluid (CSF), where levels of AA, DHA, and other FAs were increased in the acute period post-TBI (<4 days) [134,135]. CSF levels of AA-derived F2-isoprostane were greatly increased 24 h after injury in paediatric severe TBI, indicating increased lipid peroxidation that was correlated with neuronal damage, as assessed by the marker NSE [136]. Additionally, higher CSF levels of F2-isoprostane have been found to be associated with worse outcomes [137]. This highlights the role of oxidative stress in the pathological cascades taking place in the secondary injury phase of TBI, and there is evidence that sex differences exist in the acute lipid oxidative stress response after TBI, where higher increases in levels of F2-isoprostane have been observed in males 24 h after injury compared to females [138]. Also, we recently showed that the levels of DHA, as well as the levels of LPC-DHA, are low in plasma in the first 3 days following TBI [139]. Interestingly, a study by Nkiliza et al. showed that AA-derived oxylipins are increased in the plasma of veterans who are carriers of the genetic apolipoprotein ɛ4 (APOE4) polymorphism and have a history of mild TBI and post-traumatic stress disorder [140].

## 6. Cellular Targets of Long-Chain O3 PUFAs

O3 PUFAs can activate a multitude of cellular targets, which can trigger a diverse range of signalling pathways [117,141]. Long-chain PUFAs such as DHA and EPA can bind to G-protein-coupled receptors (GPRs), such as GPR40 and GPR120 (also named fatty acid receptor 1 (FFAR1) and 4 (FFAR4), respectively) [142,143]. GPR40 is widely expressed in the brain, and PUFA signalling through GPR40 activates neurogenesis and has anti-nociceptive and anti-apoptotic effects [144]. GPR120 is associated with anti-inflammatory and anti-apoptotic effects, and recent evidence shows that GPR120 expression is enriched in microglia and that its activation can modulate neuroimmune responses [145,146]. DHA and AA can also act as agonists of retinoid RXR receptors, and DHA signalling through RXRα has been shown to have effects on dendritic spine density and synaptic transmission [147,148,149]. Binding to retinoid receptors leads to signalling through multiple possible combinations of heterodimers with other receptors, such as peroxisome-proliferator receptors (PPAR). O3 PUFAs can also bind to voltage-sensitive Na^+^ and Ca^2+^ channels and can open two-pore domain background K^+^ channels such as TREK-1 [150,151].

Apart from changes induced by O3 PUFAs by signalling through specific receptors, the enrichment of cellular membranes in PUFAs such as DHA has profound consequences on membrane thickness and fluidity, the dynamics of membrane subdomains, and protein mobility [152,153].

## 7. Interventions with O3 PUFAs

The potential value of long-chain O3 PUFAs such as EPA and DHA in TBI can be viewed from a therapeutic viewpoint or as a prophylactic intervention in at-risk populations. The value of O3 PUFAs as a prophylactic intervention was first reported two decades ago in a rat FPI model, where supplementation with fish oil (DHA and EPA) for the 4 weeks preceding injury alleviated some of its deleterious effects on synaptic plasticity and cognitive abilities [154]. These early observations, as well as clear efficacy signals for acutely administered DHA in traumatic spinal cord injury (SCI) [155,156], led to the suggestion that long-chain PUFAs such as DHA had potential for neuroprotection in traumatic neurological injury [141]. Over the last decade, there has been a steady accumulation of reports in a variety of animal models of TBI, including mice and rats, with different types of injury (most notably CCI, FPI, and WD models) and with a variety of O3 PUFA dosing regimens and administration protocols. Some studies analysed the effects of a combination of O3 PUFAs (such as those that administered fish oil, i.e. DHA and EPA), others the effects of specific O3 PUFAs (most notably, DHA-only interventions), and others the impact of endogenous O3 PUFA status by inducing a deficit in O3 PUFAs or by increasing the production of endogenous O3 PUFAs.

This review discusses the impact of these manipulations on TBI outcome and the evidence from the preclinical and clinical studies carried out so far. Multiple experimental studies have investigated the therapeutic potential of O3 PUFAs as neuroprotective interventions in TBI (summary in Table 1), and the data indicate that an O3 PUFA intervention can have an overall wide therapeutic and prophylactic scope. Observations from these studies will be discussed below, with a particular focus on the experiments that have immediate translational potential due to the mode of administration being similar to what could be implemented by emergency and trauma services in human TBI (e.g. in the “golden hour” after injury).

### 7.1. Effects of Acute Single or Repeated Interventions

A few studies have investigated the effects of a single acute O3 PUFA administration following TBI. DHA (55 mg/kg) administered by gavage after CCI injury led to improved neurological function and spatial learning and memory, and also reduced cerebral oedema and hippocampal oxidative stress [157]. Similarly, after CCI in rats and mice, a single intravenous DHA administration (250 nmol/kg or 500 nmol/kg) was also associated with improved neurological function and reduced cerebral oedema, as well as improved forelimb asymmetry and reduced BBB disruption, tissue loss, and microglial and astrocyte activation [158,159].

Other studies have adopted a multiple-dose approach, where several O3 PUFA doses were administered following TBI for a number of days post-injury. DHA administered orally after CCI injury led to improvements in hippocampal function and in multiple behavioural assessments (measuring spatial learning and memory, motor activity and fine locomotor coordination, balance, and vestibulomotor function) at a dose of 200 mg/kg after injury and every 12 h, for a period of 3 days [160]. Additionally, DHA administration reduced apoptosis in the brain in a dose-dependent manner at daily doses of 370 and 740 mg/kg (intragastric, started 30 min after injury and continued daily for two weeks thereafter) [161].

Multiple-dose intraperitoneal injections of DHA and/or other O3 PUFAs have also been one of the treatment regimens used in some studies. At a dose of 16 mg/kg DHA, in rat CCI, there was a reduction in TBI-induced cerebral oedema, neuronal apoptosis, axonal injury, lesion size, white matter damage, endoplasmic reticulum stress responses, autophagy responses, and astrocyte reactivity, and also a shift in microglia morphology from amoeboid to a surveillant phenotype, and improved neurological function and cognitive performance in a multitude of tests [162,163,164,165,166]. A dose of 12 mg/kg/day DHA, starting one day after mouse CCI and continued for 20 days until the end of the study, showed no benefits in regard to balance and motor coordination or TBI-induced neuroinflammation, and no increase in neurogenesis in the subventricular zone or decrease in secondary dopaminergic neuronal loss in the midbrain [167]. A higher dose of 50 mg/kg in mouse CCI, initiated immediately after injury and continued daily for 8 days, showed that DHA had some neuroprotective effects, but overall did not significantly improve inflammation or brain function within the first week post-injury [168]. Similarly, a regime of intraperitoneal administration of 3 mg/kg DHA and 7 mg/kg EPA, first administered 2 h after CCI in mice and continued daily for 14 days, did not lead to longer-term improvements over 30 days post-injury regarding neuronal survival, cell proliferation, learning, and memory, but a different regimen, combining these injections with a dietary supplementation with fish oil (for a dietary final concentration of 4%), did show some improvements in spatial learning and memory, reduced striatal neuronal loss, and increased production of neural progenitor cells, oligodendrogenesis, and angiogenesis in the striatum and cortex [169]. Chen and colleagues reported reduced apoptotic markers, microglia activation, and cerebral oedema, and improved neurological function in rats with WD injury after DHA administration (30 min after injury and repeated for 7 days) [170,171,172]. Using a similar protocol in WD in mice, Wu et al. also reported a reduction in apoptosis and inflammation markers, and an improvement in neurological outcome [173].

**Table 1:** Overview of results in studies investigating the effects of O3 PUFA-based interventions in animal models of TBI.

Reference	Animal Model	Omega-3 Administration	Effects on Neuropathological Changes and Brain Marker Expression *		Effects on Behaviour *	
Wu et al., 2004 [154]	Male Sprague-Dawley rats, FPI	Diet with 8% fish oil (12.4% DHA and 13.5% EPA of total fat content (vs. 0.9% DHA and 1% EPA) for 4 weeks prior and 1 week after injury	7 d	↑ BDNF, synapsin I, CREB ↓ Protein oxidation (protein carbonyl)	5–7 d	↑ Spatial learning and memory (MWM)
Wu et al., 2007 [174]	Sprague-Dawley rats, FPI	Diet with 8% fish oil (12.4% DHA and 13.5% EPA of total fat content) (vs. 0.9% DHA and 1% EPA) for 4 weeks before injury	7 d	↑ Sir2, AMPK, p-AMPK, uMtCK ↓ Protein oxidation (protein carbonyl)		
Bailes et al., 2010 [175]	Adult male Sprague-Dawley rats, M-WD	10 or 40 mg/kg DHA by gavage 1 day after injury and daily after	30 d	↓ APP, caspase-3		
Mills et al., 2011 [176]	Adult male Sprague-Dawley rats, M-WD	10 or 40 mg/kg of fish oil (6 mg/kg or 24 mg/kg, DHA and EPA) by gavage, 1 day after injury and daily after	30 d	= NF-M, CytC ↓ APP, caspase-3		
Mills et al., 2011 [177]	Adult male Sprague-Dawley rats, M-WD	3, 12, or 40 mg/kg DHA by gavage for 30 days prior to injury	7 d	↓ APP, CD68, caspase-3	14 d	↑ Spatial learning and memory (MWM)
Wu et al., 2011 [178]	Sprague-Dawley rats, FPI	Diet with 1.2% DHA of total diet content (vs. standard †) after surgery and daily after	12 d	↑ BDNF, CaMKII, SYN1, CREB, SOD, Sir2, iPLA2, STX-3 ↓ 4-HNE	8–12 d	↑ Spatial learning and memory (MWM)
Ying et al., 2012 [179]	14-week-old male Sprague-Dawley rats, FPI	Diet with 0.5% flaxseed oil, 1.2% DHA, and 0.24% EPA of total diet content (480 mg/kg/day DHA) (vs. 0%); initiated on pregnant dams and carried on in pups daily after weaning	7 d	↑ BDNF, p-TrkB, p-CREB, iPLA2, STX-3 ↓ 4-HNE		
Pu et al., 2013 [180]	3-month-old male C57BL/6J mice, CCI	15 g/kg fish oil for 2 months before injury	35 d	↑ MBP, cell viability (Nissl staining), signal conduction and myelinated axon preservation (compound action potential) = Lesion size (Nissl staining) ↓ Iba1, IL-1α, IL-1β, TNF-α, COX-2, iNOS	≤14 d 22–26 d	↑ Motor function (WH) ↑ Spatial learning and memory (MWM)
					≤35 d	↑ Sensorimotor coordination (G) ↓ Locomotor asymmetry (C)
Russell et al., 2013 [181]	17-day-old male Long-Evans rats, CCI	Diet with 70 g/kg soybean oil (vs. 66.5 g/kg safflower oil and 3.5 g/kg soybean oil); initiated on pregnant dams and carried on in pups daily after weaning	1 d	↑ MMP9 = CCL2, GFAP, MMP9, TIMP-1	1 d ≤28 d	= Locomotor function (FPA) = Locomotor asymmetry (C)
			28 d	= Lesion size (cresyl violet staining)		
		Diet with 70 g/kg soybean oil (vs. 66.5 g/kg safflower oil and 3.5 g/kg soybean oil); initiated on pregnant dams and carried on in pups daily for two litters	1 d	↑ CCL2, TIMP-1 = GFAP, MMP9	1 d ≤28 d	↑ Locomotor function (FPA) ↓ Locomotor asymmetry (C)
			28 d	= Lesion size (cresyl violet staining)		
Wang et al., 2013 [182]	Male Sprague-Dawley rats, FPI	Diet with 6% fish oil (0.4% ALA, 0.6% DHA, and 0.9% EPA of total diet content) (vs. soybean oil; 0.7% ALA and 0% DHA and EPA) 4 weeks before injury onwards	14 d	= Neuronal survival (cresyl violet staining)	10–14 d	= Spatial learning and memory (MWM)
Wu et al., 2013 [183]	Sprague-Dawley rats, FPI	Diet with 1.2% DHA of total diet content (vs. standard †) after surgery and daily after	12 d	↑ Acox1, 17β-HSD4, Sir2, iPLA2, STX-3, BDNF, p-TrkB ↓ 4-HHE	8–12 d	↑ Spatial learning and memory (MWM)
Agrawal et al., 2014 [184]	17-week-old male Sprague-Dawley rats, FPI	Diet with 0.48% flaxseed oil, 1.2% DHA, and 0.24% EPA of total diet content (vs. 0% flaxseed oil, DHA, and EPA); initiated on pregnant dams and carried on in pups daily after weaning	7 d	↑ p-AMPK, COII, PGC-1α, SOD2, TFAM, BDNF, p-TrkB, p-CREB, NPY1R ↓ 4-HNE	7 d	↓ Anxiety (EPM)
Begum et al., 2014 [162]	Male Sprague-Dawley rats, CCI	16 mg/kg DHA intraperitoneally 5 min after surgery and daily after	3 d	= GRP-78 ↓ p-eIF2α, IRE1α, XBP1, ATF4, CHOP, ubiquitin, APP, p-Tau	≤5 d	↑ Motor coordination & balance (BB), fine locomotor coordination (BW)
			7 d	= GRP-78 ↓ p-eIF2α, IRE1α, XBP1, ATF4, CHOP, ubiquitin, APP, p-Tau		
			21 d	= CHOP, GRP-78 ↓ p-eIF2α, IRE1α, XBP1, ATF4, APP, p-Tau		
Desai et al., 2014 [185]	3/4-month-old C57BL/6J mice, CCI	Diet with 2.5% ALA and 0.9% DHA of total diet content (vs. 0.09% ALA and 0% DHA); initiated on pregnant dams two generations prior and carried on in pups daily after weaning	1 d	↓ αII-spectrin breakdown products	5 d	↓ Anxiety (OF)
			7 d	↑ Synapsin I	7 d	↑ Memory (NOR)
			N/A	↑ NeuN	≤7 d	↑ Fine locomotor coordination (BW), vestibulomotor function (R)
Russell et al., 2014 [186]	17-day-old male Long-Evans rats, CCI	Fish oil (1.34 g/kg DHA and 2.01 g/kg EPA) (vs. soybean oil), by gavage 30 min before injury and daily after	1 d	= CCL2, GFAP ↓ MMP9	≤7 d	↑ Fine locomotor coordination (BW)
			4 d	= CCL2, GFAP, MMP9		
			7 d	↓ IgG		
Tyagi et al., 2014 [187]	14-week-old male and female Sprague-Dawley rats, FPI	Diet with 1.2% DHA and 0.24% EPA of total diet content (vs. 0%); initiated on pregnant dams and carried on in pups daily after weaning	7 d	↑ NPY1R, BDNF, GAP-43, STX-3, iPLA2 = Nogo-A ↓ MAG, 4-HNE, s-PLA2	7 d	↓ Anxiety (EPM)
Wu et al., 2014 [188]	Male Sprague-Dawley rats, FPI	Diet with 1.2% DHA of total diet content (vs. standard †) after surgery and daily after	14 d	↑ BDNF, p-TrkB, FADS2, 17β-HSD4 ↓ 4-HNE, 4-HHE	8–12 d	↑ Spatial learning and memory (BM)
Harvey et al., 2015 [163]	Male Sprague-Dawley rats, CCI	16 mg/kg DHA intraperitoneally 5–15 min after injury and daily after	3 d	↑ CD206, neuronal survival (Fluoro Jade-C) = Iba1, CHOP, IL-1β ↓ CD16/32, LAMP1, NF-κB, TNF-α		
			7 d	↑ CD206, neuronal survival (Fluoro Jade-C) = Iba1, CD16/32, CHOP, NF-κB		
			21 d	↑ CD206, neuronal survival (Fluoro Jade-C) = Iba1 ↓ CD16/32		
Desai et al., 2016 [189]	3/4-month-old C57BL6/N male mice, CCI	Diet with 3.08% ALA of total fat content (vs. 0.04%); initiated on pregnant dams and carried on in pups daily after weaning	4 h	= IL-6, IL-10, CCL2, CD16, CD32, CD206, Arg1, Ym1/2 ↓ TNF-α, IL-1β	≤7 d 21 d	↑ Fine locomotor coordination (BW), vestibulomotor function (R) ↑ Memory (FC)
			1 d	= TNF-α, IL-1β, IL-10, CD16, CD32, Arg1, Ym1/2 ↓ IL-6, CCL2, CD206		
			3 d	= Iba1 ↓ GFAP		
			4 d	= TNF-α, IL-1β, IL-6, IL-10, CCL2, CD16, CD32, CD206, Arg1, Ym1/2		
Lucke-Wold et al., 2016 [164]	Young adult male Sprague-Dawley rats, single bTBI	16 mg/kg DHA intraperitoneally 5 min after injury and every other day after for 2 weeks	1 d	↓ GADD34, p-JNK, CHOP		
	Young adult male Sprague-Dawley rats, repeated bTBI		21 d	↓ PHF, p-Tau, BiP, p-GSK-3β	15–20 d	↑ Spatial learning and memory (MWM)
Schober et al., 2016 [190]	17-day-old male Sprague-Dawley rats, CCI	Diet with 0.1% DHA of total diet content (vs. soybean oil) or 1.8% DHA of total fat content; initiated on pregnant dams 1 day before injury and carried on in dams and pups daily after weaning	1 d	= TNF-α, IL-1β, CCL2, IL-6, IL-10, IL-2, NOx (hippocampus) ↓ NOx (cortex)	12 d 14 d 35 d	= Vestibulomotor function (R) = Memory (NOR) = Vestibulomotor function (R)
			2 d	↑ IL-6, IL-10 and IL-2 (hippocampus) = IL-6, IL-10 and IL-2 (cortex), TNF-α, IL-1β, CCL2, NOx	41–47 d	↑ Spatial learning and memory (MWM)
			3 d	= Lesion size (H&E)		
			12 d	= WM damage (MRI) ↓ Cerebral oedema (MRI), axonal injury (MRI)		
			28 d	= Axonal injury (MRI), WM damage (MRI)		
			50 d	↓ Lesion size (H&E)		
Butt et al., 2017 [191]	84–96-day-old male Sprague-Dawley rats, mFPI	Diet with 0.92% DHA of total fat content (vs. 0%) initiated 28 days before injury	23–24 d	= IL-6, IL-1β, microglia/macrophage activation (^[3H]^PK11195 autoradiography) ↓ TNF-α, IL-10	15–18 d	= Spatial learning and memory (MWM)
					22 d	= Sensory sensitivity (WNT)
		Diet with 0.92% DHA of total fat content (vs. 0%) initiated after injury	23–24 d	= IL-6, IL-1β ↓ TNF-α, IL-10, microglia/macrophage activation (^[3H]^PK11195 autoradiography)	15–18 d	= Spatial learning and memory (MWM)
					22 d	= Sensory sensitivity (WNT)
Chen et al., 2017 [170]	Adult male Sprague-Dawley rats, F-WD	2 mL/kg DHA † intraperitoneally 30 min after surgery and daily after	1 d	= Cerebral oedema (water content)	1–3 d	= Neurological function (mNSS)
			3 d	↑ SIRT1 = GFAP, nuclear HMGB1 ↓ Iba1, TNF-α, IL-1β, IL-6, IFN-γ, cytoplasmic HMGB1, cleaved caspase-3, Bax, p65, p-IκB, TLR4, cerebral oedema (water content), apoptosis (TUNEL)	7 d	↑ Neurological function (mNSS)
			7 d	↓ Cerebral oedema (water content)		
Lin et al., 2017 [192]	8-week-old male Sprague-Dawley rats, CCI	Diet with 23.8% DHA and 5.3% EPA of total fat content (vs. <0.02% DHA and <0.02% EPA); initiated on pregnant dams and carried on in pups daily after weaning	8 h	↓ IL-1β, IL-18, IL-6, caspase-1, cleaved caspase-3, apoptotic cells (TUNEL), cerebral oedema (water content), lesion size (imaging)	≤5 d	↑ Motor coordination & balance (BB)
					≤15 d	↑ Spatial learning and memory (MWM)
Pu et al., 2017 [169]	10/12-week-old male C57BL/6J mice, CCI	3 mg/kg DHA and 7 mg/kg EPA intraperitoneally 2 h after injury and daily for 14 days	35 d	= NeuN (cortex, striatum, hippocampus), cell proliferation (cortex, striatum, hippocampus) (BrdU)	29–35 d	= Spatial learning and memory (MWM)
		Fish oil for 4% O3 PUFA of total diet content (vs. 0.36%) 1 day after injury and daily for 35 days	35 d	↑ NeuN (striatum), cell proliferation (cortex, striatum) (BrdU) = NeuN (cortex, hippocampus), cell proliferation (hippocampus) (BrdU)	29–35 d	↑ Spatial learning and memory (MWM)
Zhu et al., 2017 [161]	8-week-old male and female Sprague-Dawley rats, LFP	370 or 740 mg/kg DHA intragastrically 30 min after injury and daily after	15 d	↑ Bcl-2 ↓ caspase-3, Bax	≤15 d	↑ Fine locomotor coordination (BW)
					16–17 d	↑ Spatial learning and memory (MWM)
Chen et al., 2018 [171,172]	Adult male Sprague-Dawley rats, F-WD	2 mL/kg omega-3 PUFA † intraperitoneally 30 min after injury and daily for 7 days	3 d	↑ Bcl-2, CD206, IL-10, SIRT1 = HMGB1 (in astrocytes) ↓ Cleaved caspase-3, Bax, Iba1, CD16, TNF-α, IL-1β, IL-6, HMGB1 (neurons and microglia), p65, apoptotic cells (Nissl staining, TUNEL), BBB permeability (Evans blue), cerebral oedema (water content)	≤14 d	↑ Neurological function (mNSS), vestibulomotor function (R)
			7 d	↑ LC3, LC3-II, beclin-1, ATG-3, ATG-7, p62, HO-1, NQO-1, UGT1A1, SIRT1 ↓ ROS, cerebral oedema (water content)		
			14 d	↓ Cerebral oedema (water content)		
Figueiredo et al., 2018 [193]	5/6-week-old male Sprague-Dawley rats, CCI	1500 nmol/kg ALA subcutaneously 30 min, 3 days and 7 days after injury	10 d	↓ Lesion size (stereological analysis)	30 d	↓ Anxiety (OF)
			30 d	↑ GAD 67 (in interneurons) = Neuron count (Nissl staining) ↓ lesion size (stereological analysis)		
Ghazale et al., 2018 [167]	7/8-week-old male C57BL/6 mice, CCI	12 mg/kg DHA intraperitoneally 1 day after injury and daily after	26 d	= DCX, Iba1, GFAP, TH, GFAP, αII-spectrin breakdown products	≤24 d	= Vestibulomotor function (R)
					24 d	= Locomotor coordination (PC)
Tang et al., 2018 [165]	Male Sprague-Dawley rats, CCI	16 mg/kg DHA intraperitoneally 30 min after injury and daily after	1 d	↓ TLR4, p65, IL-1β, TNF-α, CD11b, GFAP, apoptotic cells (TUNEL), cerebral oedema (water content)	≤5 d	↑ Fine locomotor coordination (BW), vestibulomotor function (R), neurological function (NSS)
Yin et al., 2018 [166]	Adult male Sprague-Dawley rats, CCI	16 mg/kg DHA intraperitoneally 10 min after injury and daily after	3 d	= Cathepsin D, p62, Lamp2, hippocampus volume (MRI) ↓ Lamp1, lesion volume (MRI), WM damage (MRI)	14–20 d	↑ Spatial learning and memory (MWM)
			7 d	= p62, Lamp1, Lamp2, cathepsin D		
Zhu et al., 2018 [194]	7-week-old male Wistar rats, FPI	370, 555 or 740 mg/kg DHA intragastrically 30 min after injury and daily after	1 d	↑ Bcl-2, HO-1, NQO-1, Nrf2, GPx, SOD ↓ caspase-3, Bax, MDA, apoptotic cells (TUNEL), cerebral oedema (water content)	≤8 d	↑ Spatial learning and memory (MWM)
					≤21 d	↑ Neurological function (NSS)
Ataizi et al., 2019 [195]	Adult male Wistar rats, M-WD	300 mg/kg omega-3 by gavage for 14 days prior to injury	0 d **	↓ CytC, caspase-3		
Schober et al., 2019 [196]	17-day-old Sprague-Dawley rats, CCI	Diet with 0.1% DHA of total diet content (vs. 0.1% soybean oil) or 3.3% DHA of total fat content; initiated on milking dams after pups received injury and carried on in pups after weaning. Intraperitoneal injection of 100–150 mg/kg DHA was also administered 30 min after injury.	1 d	↓ NOx	14 d	↑ Memory (NOR)
			3 d	= Iba1 (hippocampus), GFAP (cortex, hippocampus) ↓ Iba1 (cortex), CD68 (cortex)		
			7 d	= Iba1 (cortex, hippocampus), GFAP (cortex, hippocampus), CD68 (cortex)		
Liu et al., 2020 [158]	Young adult male Sprague-Dawley rats, CCI	250 nmol/kg DHA intravenously 30 min after injury	1 d	↓ Cerebral oedema (MRI)	≤7 d	↑ Neurological function (mNSS) ↓ Locomotor asymmetry (C)
			3 d	↓ Cerebral oedema (MRI)		
			7 d	↑ RECA-1, CD31, occludin = GFAP ↓ IgG, AQP-4, MMP9 (in astrocytes), cerebral oedema (MRI)		
Reyes et al., 2020 [197]	3-/4-month-old male C57BL/6 mice, CHIMERA	Diet with 3.8% ALA of total fat content (vs. 0.04%); initiated in pregnant dams and continued in pups daily after weaning	3 m	= WM damage (MRI)		
Thau-Zuchman et al., 2020 [159]	10-/12-week-old male and female CD1 and C57BL/6 mice, CCI	500 nmol/kg DHA intravenously 30 min after surgery	7 d	↑ NeuN, cell proliferation (BrdU) ↓ Iba1, GFAP, APP, 8-OHG, lesion size (H&E and MRI)	≤28 d	↑ Neurological function (mNSS)
			14 d	= Lesion size (H&E and MRI)		
			28 d	= Iba1, GFAP, lesion size (H&E and MRI)		
Zhang et al., 2020 [198]	3-/4-month-old male C57BL/6 mice, CCI	15 g/kg fish oil † by gavage for 2 months prior to injury	7 d	↑ ZO-1, occludin, glymphatic system function (radioisotope clearance assay) = Lesion size (H&E) ↓ Aβ42, AQP-4, BBB permeability (Evans blue)	≤7 d	↑ Neurological function (mNSS), vestibulomotor function (R)
Zhu et al., 2020 [160]	3-month-old male C57BL/6 mice, CCI	200 mg/kg DHA by gavage immediately after injury and every 12 h for 3 days	1–3 d	↓ p-JNK, p-Tau	≤14 d	↑ Fine locomotor coordination (BW), motor coordination & balance (BB), spatial learning and memory (MWM), vestibulomotor function (R)
			14 d	↑ Hippocampal long-term potentiation		
Zhu et al., 2020 [157]	10-/12-week-old male Sprague-Dawley rats, CCI	55 mg/kg DHA by gavage immediately after injury	3 d	↑ Nrf2, HO-1, NQO-1 ↓ NOX2, cerebral oedema (water content)	7–10 d	↑ Spatial learning and memory (MWM)
					≤21 d	↑ Neurological function (NSS)
Desai et al., 2021 [199]	3-month-old male and female C57BL/6NCrl mice, CHIMERA	Diet with 3.37% ALA of total fat content (vs. 0.05%); initiated in pregnant dams and carried on in pups after weaning	60 d	↓ Iba1, GFAP, axonal degeneration (silver staining)	60 d	↑ Spatial learning and memory (MWM), visual function (VEP and ERG)
Schober et al., 2021 [200]	17-day-old male Sprague-Dawley rats, CCI	Diet with 0.1% DHA of total diet content (vs. 0.1% soybean oil) or 1.8% DHA of total fat content; initiated in pregnant dams 1 day before injury and carried on in dams, and in pups after weaning	2 d	= iNOS, IL-18, IL-18rap (hippocampus), TNF-α, IL-1β (cortex), TGF-β ↓ IL-18rap (cortex), IL-1β (hippocampus)		
			3 d	= iNOS, IL-18, IL-18rap, TNF-α, IL-1β, TGF-β		
			3–50 d	= TSPO		
Shi et al., 2022 [168]	C57/BL mice CCI	50 mg/kg DHA intraperitoneally after injury and daily for 8 d	8 d	↑ eNOS, FGF21, VEGFR, β-catenin, MDM2 = IL-1β, IL-6, IL-10, TNF-α, cleaved PARP-1, HIF-1α, Wnt ↓ GSK-3β, p53	7 d	= Spatial learning and memory (MWM)
Wu et al., 2023 [173]	Adult male C57BL/6J mice, F-WD	2 mL/kg omega-3 PUFA † intraperitoneally 30 min after injury and daily after	3 d	↑ PPARγ ↓ TNF-α, IL-1β, IL-6, NF-κB, RIP1, RIP3, MLKL, apoptotic cells (TUNEL), cerebral oedema (water content)	3 d	↑ Neurological function (mNSS)

* Changes observed in the last two columns refer to significant changes in O3 PUFA-treated TBI groups relative to control TBI groups. † Exact O3 PUFA formulation, dose and/or concentration not specified. ** Time of analysis not clear in the text. **Animal models:** bTBI: blast-related traumatic brain injury, CCI: controlled cortical impact, CHIMERA: closed-head impact model of engineered rotational acceleration, FPI: fluid percussion injury, F-WD: Feeney’s weight drop, LFP: lateral fluid percussion, mFPI: midline fluid percussion injury, M-WD: Marmarou’s weight drop. **Omega-3 administration:** ALA: α-linolenic acid, DHA: docosahexaenoic acid, EPA: eicosapentaenoic acid, PUFA: polyunsaturated fatty acid. **Brain markers:** Aβ42: 42-amino acid amyloid beta peptide, Acox1: acyl-CoA oxidase 1, APP: beta-amyloid precursor protein, AQP-4: aquaporin-4, AMPK: AMP-activated protein kinase, Arg1: arginase 1, ATF4: activating transcription factor 4, ATG-3/7: autophagy-related genes 3 or 7, Bax: Bcl-2-associated X protein, BBB: blood–brain barrier, Bcl-2: B-cell lymphoma-2, BDNF: brain-derived neurotrophic factor, BiP: binding immunoglobulin protein, BrdU: bromodeoxyuridine, CaMKII: Calcium–calmodulin-dependent protein kinase II, CHOP: C/EBP homologous protein, CCL2: CC-motif ligand 2, CD11b/16/31/32/68/206: cluster of differentiation 11b, 16, 31, 32, 68, or 206, COII: cytochrome C oxidase II, COX-2: cyclooxygenase-2, CREB: cAMP response element-binding protein, CytC: cytochrome C, DCX: doublecortin, eIF2α: eukaryotic initiation factor 2, eNOS: endothelial nitric oxide synthase, FADS2: fatty acid desaturase 2, FGF21: fibroblast growth factor 21, GADD34: growth arrest and DNA damage-inducible protein 34, GAD 67: glutamic acid decarboxylase 67, GAP-43: growth-associated protein 43, GFAP: glial fibrillary acidic protein, GPx: glutathione peroxidase, GRP-78: glucose-regulated protein-78, GSK-3β: glycogen synthase kinase-3β, HIF-1α: hypoxia-inducible factor-1 alpha, HMGB1: high-mobility group box 1, HO-1: heme oxygenase-1, H&E: haematoxylin and eosin, Iba1: ionized calcium-binding adapter molecule 1, IFN-γ: interferon-gamma, IgG: immunoglobulin G, IL-1α/1β/2/6/10/18/18rap: interleukin 1 alpha, 1 beta, 2, 6, 10, 18, or 18 receptor accessory protein, iNOS: cytokine-inducible nitric oxide synthase, iPLA2: calcium-independent phospholipase A2, IRE1α: inositol requiring enzyme 1 alpha, IκB: inhibitor of NF-κB, JNK: c-Jun N-terminal kinase, LAMP1/2: lysosomal associated membrane protein 1 or 2, LC3: microtubule-associated protein 1 light chain 3, MAG: myelin-associated glycoprotein, MBP: myelin binding protein, MDA: malondialdehyde, MDM2: mouse double minute 2, MLKL: mixed lineage kinase domain-like, MMP9: matrix metallopeptidase 9, MRI: magnetic resonance imaging, NeuN: neuronal nuclear protein, NF-κB: nuclear factor kappa B, NF-M: neurofilament M, NOx: nitrates/nitrites, NOX2: NADPH oxidase 2, NQO-1: NAD(P)H quinone dehydrogenase 1, NPY1R: neuropeptide Y1 receptor, Nrf2: nuclear factor erythroid 2–related factor 2, p-: phosphorylated, PARP-1: poly(ADP-ribose) polymerase-1, PGC-1α: peroxisome proliferator-activated receptor gamma coactivator 1 alpha, PHF: paired helical filament, iPLA2: calcium-independent phospholipase A2, PPARγ: peroxisome proliferator-activated receptor gamma, RECA-1: rat endothelial cell antigen-1, RIP1/3: receptor-interacting protein 1 or 3, ROS: reactive oxygen species, s-: secreted, Sir2: silent information regulator 2, SIRT1: sirtuin 1, SOD/2: superoxide dismutase, or 2, STX-3: syntaxin-3, SYN1: synapsin I, Tau: tubulin associated unit, TFAM: mitochondrial transcription factor A, TGF-β: transforming growth factor-β, TH: tyrosine hydroxylase, TIMP-1: tissue inhibitor of metalloproteinases-1, TLR4: toll-like receptor 4, TNF-α: tumour necrosis factor-alpha, TrkB: tyrosine receptor kinase B, TSPO: translocator protein, TUNEL: terminal deoxynucleotidyl transferase dUTP nick end labelling, UGT1A1: UDP Glucuronosyltransferase family 1 member A1, uMtCK: ubiquitous mitochondrial creatine kinase, VEGFR: vascular endothelial growth factor receptor, WM: white matter, Wnt: wingless-related integration site, XBP1: X-box binding protein 1, ZO-1: zonula occludens-1, 4-HHE: 4-hydroxy-2-hexenal, 4-HNE: 4-hydroxynonenal, 8-OHG: 8-hydroxyguanosine, 17β-HSD4: 17 beta-hydroxysteroid dehydrogenase 4. **Behavioural tests:** BB: beam balance, BM: Barnes maze, BW: beam walk, C: cylinder test, EPM: elevated plus maze, ERG: electroretinogram, FC: fear conditioning, FPA: force-plate actometer, G: grid walk, mNSS: modified neurological severity score, MWM: Morris water maze, NOR: novel object recognition, NSS: neurological severity score, OF: open field, PC: pole climbing, R: rotarod, VEP: visual evoked potential, WH: wire hanging, WNT: whisker nuisance task.

### 7.2. Effects of Dietary Supplementations

Preclinical studies evaluating the impact of changes in dietary intake of O3 PUFAs on TBI outcome have used approaches that either create deficiencies or increase intake through supplementation. DHA supplementation (10 or 40 mg/kg/day) for 30 days after WD injury in rats helped relieve the axonal deficits seen after injury [175], and similar effects were reported with a mixture of EPA and DHA [176]. A diet high in DHA (1.2% of diet content) showed improved learning abilities in rats after FPI two weeks after injury, and also improved hippocampal parameters, as indicated by an increase in neurogenesis and plasticity and a reduction in oxidative stress [178,183,188].

Other studies have investigated the neuroprotective effects of O3 PUFAs by adopting regimens where supplementation was initiated prior to injury. Dietary supplementation with O3 PUFA for 2 weeks before WD injury in rats reduced the increase in caspase-3, cytochrome c (CytC), and serum NSE post-injury [195]. The effects of supplementation with fish oil for 1 month prior to injury was investigated in three studies; dietary supplementation with 8% fish oil was associated with an improvement in hippocampal neuronal survival and growth, a restoration of energy-sensing and genomic stability systems (such as silent information regulator 2 (Sir2) and AMP-activated protein kinase (AMPK)), a reduction in oxidative damage, and an improvement in learning and memory abilities [154,174], whereas there were no effects on hippocampal neuronal survival and function in a study with 6% fish oil supplementation [182]. Similarly, dietary prophylactic administration of DHA (at doses of 3, 12, or 40 mg/kg/day for 1 month prior to injury) in a rat WD model had protective effects on the corticospinal tracts and medial lemnisci, with reduced markers of cellular death and axonal injury, and this tissue protection was linked to improved spatial memory at 7 days post-injury [177]. An extended prophylactic administration of O3 PUFAs for 2 months prior to injury by supplementation with 15 g/kg fish oil in mice with CCI also showed improved neurological recovery for both sensory and cognitive deficits and reduced inflammation, microglia activation, white matter injury, and hippocampal neuronal loss [180]. Using the same regime of supplementation in mice with CCI, Zhang and colleagues showed that the intervention protected against changes in glymphatic clearance and in aquaporin-4 (AQP-4) expression post-injury [198]. Butt and colleagues administered a DHA-enriched diet (for 1% final content) in an mFPI rat model for 28 days prior to injury or for 24 days after injury, and showed that both types of administrations reduced the injury-induced increase in some inflammatory cytokines, but neither improved sensory sensitivity or spatial learning and memory, and only post-injury DHA administration reduced the neuroinflammatory reaction (as assessed with PET imaging using a marker for microglia activation) [191]. Finally, there is experimental evidence that O3 PUFA supplementation synergises with exercise, with the combination achieving optimum results, as observed in a TBI model in rats [183]. This is an important observation that suggests that a beneficial impact of O3 PUFA interventions may synergise with neurological rehabilitation in patients.

It is interesting to note that there are some dietary modification studies where the dietary O3 FA changes were implemented in pregnant dams [179,181,184,185,187,189,190,192,197,199,200], sometimes more than one generation preceding the breeding of animals used in the injury experiments [185], resulting in changes to the O3 status prior to initiation of the injury and thus having more relevance from a perspective of impact of deficiency or of prophylactic value. Using the closed-head impact model of engineered rotational acceleration (CHIMERA) in mice, a study found that deficiency in O3 PUFAs, where a diet with adequate or deficient ALA content (3.8% and 0.04% in content, respectively) was administered in animals from before birth and after injury, showed no effects on white matter damage more than 3 months after TBI [197]. This is in contrast with previous similar studies, which suggested that sufficiency confers a protection of grey matter in mice [185]. Tyagi and colleagues implemented an O3-adequate diet (with 1.2% DHA and 0.24% EPA content) and compared it to a deficient diet (without DHA or EPA) in pregnant rat dams whose offspring were submitted to FPI, and noted that the deficiency worsened outcome in the elevated plus maze; decreased levels of the synaptic marker syntaxin-3, brain-derived neurotrophic factor (BDNF), and GAP-43 (a regeneration marker); and also amplified the expression of growth-inhibitory molecules and oxidative stress after injury [187]. The group also showed that the O3-adequate diet could offer resilience against metabolic perturbations created by a Western-type diet, which may affect neuroplasticity [201].

A different approach to assess the impact of an increase in tissue levels of O3 PUFAs can be based on the use of *fat-1* mice, where a genetic modification renders the animals able to synthesize O3 PUFAs endogenously from dietary O6 PUFAs. Using a WD model, Lecques and colleagues showed that these *fat-1* mice are more resilient to mild TBI, as indicated by their improved neurological outcome within the first week after injury [202], and a later study in a repetitive WD model also showed an improvement in neurological function 7 days post-injury, as well as reduced astrogliosis [203].

### 7.3. Interventional Studies in Immature Animals

Several studies have investigated the impact of interventions with O3 PUFAs in TBI occurring in juvenile animals [181,186,190,193,194,196,200].

Acute post-TBI administration was associated with reduced lesion size, cerebral oedema, and expression of markers of apoptosis and oxidative stress, and improved neurological outcomes [193,194]. Similarly, administration of a diet with 0.1% DHA (providing DHA as 1.8% of total fat) or supplementation with 15 mL/kg of fish oil was also associated with decreased lesion size and cerebral oedema, as well as reduced IgG infiltration, and reduced expression of MMP9, a key player in TBI-associated BBB disruption [186,190]. In addition, there was also a long-term reduction in axonal injury, but O3 PUFA treatment overall showed little effect on inflammatory and neuroinflammatory marker expression [186,190]. Dietary supplementation primed by an intraperitoneal DHA injection 30 min after injury led to decreased cortex microglia activation but not astrocyte activation 3 days after injury, suggesting that the fatty acid modulates microglia towards a less inflammatory profile shortly after injury [196]. This reduced microglia activation was, however, not observed 7 days after injury [196]. Moreover, some improvements in cognitive function were also observed in juvenile animals treated with O3 PUFAs, including less severe hindlimb deficits 7 days after injury and improved memory [186,190,194,196].

Some of these effects are in contrast with observations made in a study that carried out dietary changes in pregnant dams and in rat pups after weaning [181], creating various levels of DHA deficit. In this study, lesion size and several markers of BBB disruption, inflammation, and neuroinflammation were unaffected by DHA levels in brain tissue, although sensorimotor outcomes were improved at higher DHA levels [181].

### 7.4. Interventional Studies in Humans

**Figure 1:**
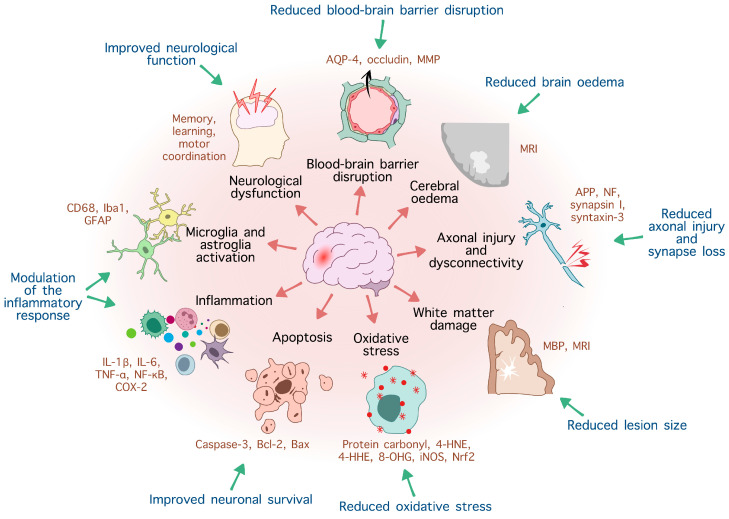
Neuropathological aspects of TBI are alleviated after omega-3 PUFA interventions. TBI leads to a number of changes in brain structure and function; some key changes are depicted in the figure (black). O3 PUFA compounds such as DHA or EPA modulate these injury-induced effects (blue), as indicated by preclinical studies in animal models of TBI, where a combination of histological markers, neuroimaging techniques, and behavioural analyses (brown) have been used to study each individual component (list of markers not exhaustive). APP: beta-amyloid precursor protein, AQP-4: aquaporin 4, Bax: Bcl-2-associated X protein, Bcl-2: B-cell lymphoma-2, CD68: cluster of differentiation 68, COX-2: cyclooxygenase-2, GFAP: glial fibrillary acidic protein, Iba1: ionized calcium-binding adapter molecule 1, IL-1β/6: interleukin 1 beta or 6, iNOS: inducible nitric oxide synthase, MBP: myelin binding protein, MMP: matrix metallopeptidase, MRI: magnetic resonance imaging, NF: neurofilament, NF-κB: nuclear factor kappa B, Nrf2: nuclear factor erythroid 2–related factor 2, TNF-α: tumour necrosis factor-alpha, 4-HHE: 4-hydroxy-2-hexenal, 4-HNE: 4-hydroxynonenal, 8-OHG: 8-hydroxyguanosine.

Overall, experimental studies point towards the neuroprotective potential of O3 PUFAs in traumatic injury, with some discrepancies between studies that are likely due to differences in dosage, type of O3 PUFAs administered, mode of administration, frequency of administration, and TBI animal model used. It is clear that the impact of these compounds on key pathophysiological mechanisms triggered by TBI (Figure 1) is significant across developmental time and animal species. DHA-only treatments or combinations of DHA and EPA reduce cerebral oedema, apoptotic death, and neuronal loss, limit axonal injury and the loss of synaptic proteins, attenuate proinflammatory microglia and astrocytic activation, reduce oxidative stress, improve myelin preservation, and increase levels of neurotrophic factors (such as BDNF). There is also some evidence that treatment with DHA has a beneficial impact on the abnormal protein aggregation triggered by TBI, affecting proteins such as β-amyloid precursor protein (APP) and tubulin-associated unit (tau) [160,162], which are associated with tauopathies such as AD or ALS.

A double-blind placebo-controlled randomised controlled trial (RCT) was carried out in American football players that were administered DHA at doses of 2, 4, or 6 g/day to assess the impact of this intervention on serum NFL, a marker of TBI [204]. Over a period of 189 days, DHA supplementation significantly increased plasma DHA in a dose-dependent manner and led to an attenuated increase in serum NFL, coinciding with the active competitive season, suggesting that the prophylactic use of DHA in the high-risk population of contact sport athletes may confer neuroprotection [204]. This dose–response effect of DHA was further evaluated in another study, where higher DHA doses were associated with a higher O3I, e.g. 6 g/day leading to O3I > 8% after 8 weeks [205]. Furthermore, another study found that only 1 out of 30 elite collegiate American football athletes had an O3I within the recommended range [206], further highlighting the importance of and need for the profiling of athletes according to their O3 levels. Additionally, supplementation with DHA at a dose of 2 g/day for a period of 12 weeks in adolescents within 4 days of a sports-related concussion showed encouraging (albeit non-significant) trends towards a decrease in recovery time, where participants treated with DHA were symptom-free earlier than the placebo group (11 vs. 16 days) [207]. Similarly, use of enteral nutrition formulations containing DHA and EPA (with added vitamins and amino acids that support immune modulation) in patients with severe TBI reduced length of hospital stay, although it had no effects on inflammation marker levels or infection rates [208]. A double-blind RCT in 110 trauma patients from an intensive care unit assessed the effects of dietary supplementation with fish oil (containing a dose of 1.47 g DHA and 147 mg EPA per day) for 12 weeks on quality of life (QoL) parameters, measured by the Medical Outcomes Study 36-Item Short Form (SF-36) survey [209]; results showed that supplementation did not affect any of the eight QoL domains measured by the SF-36 survey after 12 weeks, although erythrocyte DHA levels increased in the treated group, which confirmed compliance and DHA uptake by the body [209].

In a different study design, the administration of O3 PUFAs was combined with additional components (polyphenols) for a synergistic effect; in this small study including nine participants with severe TBI, high daily doses of DHA and EPA (5.4 g DHA and 10.8 g EPA) were acutely administered alongside polyphenol supplements, and it resulted in all patients showing improvements in their GCS scores over the course of the study [210]. However, the open-label nature of the study and lack of a placebo control group reduce the robustness of the study.

Finally, other clinical studies have instead investigated other aspects of the O3 status and its link to the TBI outcome. For instance, one study found that the serum levels of specific oxylipins 3 days after injury were associated with long-term post-traumatic headache severity; they found that DHA derivatives 4-hydroxy-DHA and 19,20-epoxy-docosapentaenoate were inversely associated with headache severity, whereas LA derivative 11-hydroxy-9-epoxy-octadecenoate was positively associated with headache severity, which suggests that these DHA-derived oxylipins could be used as prognostic biomarkers for the development of chronic post-traumatic headaches [211]. Post-traumatic headache is a common and debilitating consequence of TBI that generally develops within a few days of injury and usually resolves within 2–3 months, but about 40% of sufferers have headaches persisting beyond the 3 month mark [212]. Another study found an association between loss of consciousness following a head injury and intake of O3 PUFAs, where DHA and EPA consumption was 22.7% and 35.2% lower, respectively, in the group with head injury and loss of consciousness [213].

## 8. Questions for Clinical Translation

Taken as a whole, the reviewed data suggest that the potential of O3 PUFAs in neurological traumatic injury is significant—and in particular for DHA, for which there is more information so far. O3 PUFA-based interventions could have value over a range of types of injury, and from the acute to the subacute period. However, there are still some unresolved questions that require clarification to support a successful translation to the clinic, which are addressed below.

### 8.1. Would All TBI Patients, or Just a Specific Subpopulation of TBI Patients, Respond to O3 PUFAs?

There is a need for revision of the current TBI classification, which is limited and lacks insight into the pathophysiologic mechanisms of the injury [32]. Brain biopsies obtained from living adult cases of severe TBI shortly after injury have shown there is marked heterogeneity in the pattern of tissue loss, in particular with reference to the degree of glial and neuronal loss [214]. To address this concern and improve patient stratification, an initiative was launched by the US National Institutes of Health in 2023 to improve the characterisation of the TBI injury beyond the GCS—for example, by adding pupillary reactivity, blood-based biomarkers, and imaging data, and also including potential modifiers (e.g. extracranial injuries) [215].

The data from animal studies do not yet allow for prediction of whether the efficacy of an intervention using O3 PUFAs would be limited to patients within a specific severity range only, or whether O3 PUFA supplementation would be of any benefit if administered long after injury has taken place, especially in patients with long-lasting complications such as secondary epilepsy or cognitive and psychological changes. The discussion regarding which format a human study should have is also unclear. While high-quality and adequately powered RCTs remain a foundation of evidence-based medicine, they are best suited to interventions in patients with homogeneous diagnostic categories, and they may not be able to provide optimum guidance in conditions that are mechanistically heterogeneous, such as TBI, which is known to be driven by multiple mechanisms that may partly overlap in the timeline post-injury. Instead, alternative clinical trial designs should be considered—for example, comparative effectiveness research approaches [89], or well-designed adaptive trial designs, which could increase the probability of identifying an effective therapy regime and hasten elimination of futility. It is also necessary to continue the characterization of the various individual factors that could change the response to O3 PUFA administration, such as genetic background, sex, or gut microbiome [216].

### 8.2. How Variable Is the DHA/EPA Status of Patients?

Patients with TBI may have wide interindividual variations in their O3 baseline status; this can now be easily assessed by measuring the O3I. A large systematic review including data from 298 studies showed that blood O3 PUFA levels are variable across populations; this study found that regions with high EPA+DHA blood levels (>8%) include Japan, Scandinavia, and areas with populations not fully adopting Western-style dietary habits, whereas rather low levels (≤4%) were observed in Europe, North America, Central and South America, the Middle East, Southeast Asia, and Africa [111]. The low values seen for most of the world are akin to a situation of deficiency in O3 PUFAs and may intrinsically impact the outcome of TBI, as suggested by experimental observations in mice and rats. In a recent study, we showed that, at admission to a Level 1 Trauma Centre in London, patients with TBI had an average O3I of <5% [139]. Therefore, in the first instance, an intervention with O3 PUFAs in the form of oral administration of fish oil capsules or emulsions (containing DHA and EPA), or pure DHA supplements, could improve the O3 status beyond any other neurotrauma-specific therapeutic considerations. A relative depletion in O3 PUFAs is particularly important to be addressed in children and adolescents, as these compounds have an important neurodevelopmental role, and decreased levels of DHA in the developing brain leads to deficits in neurogenesis and alterations in learning and visual function [217].

### 8.3. Which Receptors and Mechanisms Underlie the Effectiveness of DHA?

Long-chain PUFAs such as EPA and DHA have complex pharmacodynamics and a wide array of potential targets, but at present, there is little understanding regarding which receptor targets are critical for efficacy of these compounds in TBI, especially in humans. Additionally, there is still much to be understood concerning the differences between EPA and DHA [218,219]. It is important that studies continue to explore the differences between DHA and EPA, which are still incompletely characterized, as it is known that these two fatty acids may compete with each other and have different effects on the biophysical properties of membranes and cell signalling [220].

In CCI in mice, the knockout of TREK-1 did not lead to differences in outcome compared to wild-type mice, even though this channel is a target of O3 PUFAs [221]. In another example of injury of the CNS, i.e. SCI, PPARα was suggested to be a crucial mediator of the early anti-inflammatory effects of DHA [222]. More research is required to characterise the receptor or receptor combinations that are major mediators of the neuroprotective effects. The clarification of these cellular targets will also enable a refinement of other aspects of a final clinical protocol, especially for injectable preparations, i.e. the optimum time window of administration after injury, and treatment duration.

Linked to this aspect, and to the possibility of the beneficial effects of O3 PUFAs involving multiple receptors, it is important to note an ongoing debate in the field of neuroprotection research for neurotrauma regarding the targeting of single mechanisms, or the opposite, i.e. the use of pleiotropic compounds. Would the use of compounds with pleiotropic effects and multiple targets have a higher chance of success? An example of a single mechanism and target-specific neuroprotective intervention that failed in TBI is that of magnesium to block NMDA glutamate receptor-mediated toxicity [223]. Based on the rationale of the importance of excitotoxicity as an early pathological mechanism, magnesium sulphate was administered in people with moderate to severe TBI less than 8 h after injury, followed by continuous infusions for the following 5 days [223]. Results showed that patients receiving the lower dose did significantly worse than those on placebo, and there was increased mortality at the higher magnesium dose compared to the placebo group, suggesting that not only was magnesium not neuroprotective but that it was potentially harmful. In contrast, progesterone is an example of failure of a pleiotropic agent for the treatment of severe TBI. In this instance, two large multicentre clinical trials showed that there was no difference in TBI outcome at 6 months, even after intervention was initiated as early as 4 h after injury, with continued administration of the compound for up to 120 h [224,225]—and this is in spite of numerous preclinical studies showing efficacy in TBI models [226]. Thus, as reviewed by Bragge et al., despite a considerable investment of resources and effort in carrying out over 190 RCTs to evaluate the use of different interventions for the acute management of adult and paediatric TBI, the field has not yet led to clinical breakthroughs [227].

Recent years have also seen an increase in reports of intrinsic neuroprotective effects of bioactive lipid mediators derived from long-chain O3 PUFAs. Several studies demonstrated positive neuropathological and neurobehavioural effects in experimental TBI models after the acute administration of lipid mediators such as resolvins D1 and E1 (RvD1 and RvE1, respectively), docosahexaenoyl ethanolamide (DHEA), and neuroprotectin D1 (NPD1), using various administration regimens (Table 2) [228,229,230,231,232,233]. This therefore raises the question of whether the effects of PUFAs should be considered intrinsic effects or just effects as precursors of the real bioactive species in the form of downstream lipid mediators such as the SPMs (and which would involve receptors different from those targeted by the parent PUFAs). 

### 8.4. Which Other Traumatic Injuries May Benefit Apart from TBI?

The issue of whether O3 PUFAs can be developed into a preparation with neuroprotective effects is equally important in the field of SCI. Brain and spinal cord injuries are often comorbid, particularly after motor vehicle collisions or falls [234]. The secondary injury cascade after traumatic injury of the spinal cord has many physiopathological similarities with TBI, and data obtained by us and other groups have shown clear potential of DHA for neuroprotection in the acute phase of SCI [155,156,222,235]. Additionally, DHA treatment has the potential to alleviate significant complications of this injury, such as neuropathic pain [236]. Therefore, a successful neuroprotective intervention with O3 PUFAs could address complex therapeutic challenges in injury in the CNS across a wide spectrum of unmet needs.

**Table 2:** Overview of results in studies investigating the effects of O3 metabolite-based interventions in animal models of TBI.

Reference	Animal Model	Omega-3 Metabolite Administration	Effects on Neuropathological Changes and Brain Marker Expression *		Effects on Behaviour *	
Harrison et al., 2015 [228]	Adult male C57BL/6 mice, mFPI	100 ng RvD1 intraperitoneally for 3 days before injury and for 4 days after	7 d	= Ramified microglia ↓ Rod microglia	1 d	= Sleep (PRS)
					6 d	↑ Memory (NOR)
					≤7 d	↑ Vestibulomotor function (R)
		100 ng RvE1 intraperitoneally for 3 days before injury and for 4 days after	7 d	↑ Ramified microglia ↓ Rod microglia	1 d	↑ Sleep (PRS)
					6 d	= Memory (NOR)
					≤7 d	= Vestibulomotor function (R)
Bisicchia et al., 2018 [229]	Adult male Wistar rats, HCb	0.4 μg/kg RvD1 intraperitoneally after injury and every other day after	7 d	↑ NeuN, miR-146b, miR-219-1-3p = NF-κB, CD200, miR-142-5p, miR-203a, miR-21 ↓ CytC, Iba1, GFAP, TLR4, IL-6R	≤7 d	↑ Neurological function (NSS)
Berg et al., 2019 [230]	Male Sprague-Dawley rats, pTBI	50 ng NPD1 intralesional and immediately after injury	1 d	= MnSOD, 3-NT, CD11b, COX-2, NF-κB		
			3 d	= MnSOD, 3-NT, CD11b, COX-2, NF-κB, apoptotic cells (TUNEL), neuronal degeneration (Fluoro Jade-B) ↓ Lesion area (imaging)		
Ren et al., 2020 [231]	C57BL/6 mice, CCI	15 µg/kg RvD1 intraperitoneally on the day of injury and daily after	1 d	= FRP2	≤7 d	= Fine locomotor coordination (BW)
			3 d	= FRP2	3–14 d	↑ Memory (FC)
			7 d	↑ NeuN, synaptophysin, NLRP3, ASC, IL-1β, TNF-α, IL-6, MCP-1, ATP, Parkin, BDNF, GLAST, GLAST dimers, GLUT1, GLUT3 = FRP2, LC3I/II ↓ GFAP, Pink, TRX2, BBB permeability (Evans blue)		
Ponomarenko et al., 2021 [232]	3-month-old male Wistar rats, WD	10 mg/kg DHEA subcutaneously immediately after injury and daily after	7 d	↓ Iba1, IL-1β, IL-6, CD86	5 d	= Working memory (YMSA)
					6 d	↓ Anxiety (EPM)
					7 d	↑ Long-term memory (PA)
Ponomarenko et al., 2022 [233]	3-month-old male Wistar rats, WD	10 mg/kg DHEA subcutaneously immediately after injury and daily after	1 d	↑ SOD ↓ GFAP, S100B, nNOS, BDNF		
			7 d	↑ GFAP, BDNF = S100B ↓ nNOS, SOD		

* Changes observed in the last two columns refer to significant changes in O3-treated TBI groups relative to control TBI groups. **Animal models:** CCI: controlled cortical impact, HCb: hemicerebellectomy, mFPI: midline fluid percussion injury, pTBI: penetrating traumatic brain injury, WD: weight drop. **Omega-3 metabolite administration:** DHEA: N-docosahexaenoylethanolamine, NPD1: neuroprotectin D1, RvE1/D1: resolvin E1 or D1. **Brain markers:** ASC: apoptosis-associated speck-like protein containing a CARD (caspase recruitment domain), ATP: adenosine triphosphate, BBB: blood–brain barrier, BDNF: brain-derived neurotrophic factor, CD11b/86/200: cluster of differentiation 11b, 86, or 200, COX-2: cyclooxygenase-2, CytC: cytochrome C, FRP2: N-formyl peptide receptor 2, GFAP: glial fibrillary acidic protein, GLAST: glutamate aspartate transporter, GLUT1/3: glucose transporter 1 or 3, Iba1: ionized calcium-binding adapter molecule 1, IL-1β/6/6R: interleukin 1 beta, 6 or 6R, LC3: microtubule-associated protein 1 light chain 3, MCP-1: monocyte chemoattractant protein-1, miR: microRNA, MnSOD: manganese superoxide dismutase, n-: neuronal, NeuN: neuronal nuclear protein, NF-κB: nuclear factor kappa B, NLRP3: NOD-, LRR-, and pyrin domain-containing protein 3, NOS: nitric oxide synthase, SOD: superoxide dismutase, S100B: S100 calcium-binding protein B, TLR4: toll-like receptor 4, TNF-α: tumour necrosis factor-alpha, TRX2: thioredoxin 2, TUNEL: terminal deoxynucleotidyl transferase dUTP nick end labelling, 3-NT: 3-nitrotyrosine. **Behavioural tests:** BW: beam walk, EPM: elevated plus maze, FC: fear conditioning, NOR: novel object recognition, NSS: neurological severity score, PA: passive avoidance, PRS: piezo recording system, R: rotarod, YMSA: Y-maze spontaneous alternation.

### 8.5. Which O3 PUFAs Preparations Are Available?

As reviewed above, the data obtained in experimental models cover a variety of regimes of administration, from dietary supplementation to single or multiple intravenous bolus, repeated intraperitoneal administrations, or combinations of injections and oral supplementation.

The acute administration of omega-3 PUFAs may share some protective mechanisms with chronic administration, and it may be possible to enhance the value of an acute parenteral single bolus administration with exposure to a diet enriched in DHA. There is a wide range of commercially available oral preparations that contain DHA and EPA, and experimental studies support the efficacy of DHA, as well as DHA and EPA combinations, but they still do not give a clear indication of what the optimum regime and preparation should be for clinical studies in patients with TBI. However, encouraging effects have been shown from the use of specific commercially available preparations in sports-related concussions, as mentioned above [204,205], and recent reviews of nutritional interventions in TBI highlight the potential of O3 PUFA-based treatment to improve recovery [237,238].

The O3 PUFA preparations used in animal models for a parenteral regime vary widely, and in the clinic, the available O3 PUFA-containing parenteral preparations that have received regulatory approval in the last decade are developed for parenteral nutrition [239], such as Omegaven^®^, SMOFlipid^®,^ and Lipoplus^®^, and not as an injectable neuroprotectant in the acute phase post-injury. It is thus very encouraging to see the recent development of new O3 PUFA intravenous emulsions that have translational potential for the acute management of TBI [240]. The fast intervention with injectable preparations is particularly relevant in the case of severe injuries in adults and children.

As mentioned previously, it may also be possible to enhance the effect of O3 PUFAs by combining them with other compounds to achieve mechanistic synergism. This could stimulate the development of new formulations for clinical use. For example, a proprietary preparation, which is a medical multi-nutrient containing O3 PUFAs (DHA and EPA) and other compounds involved in the biosynthesis of phospholipids (which are depleted after TBI [125]), has shown significant effects in mouse CCI both for improved neurological outcome and for tissue protection [241].

## 9. Conclusions

The studies reviewed here indicate that O3 PUFAs are compounds that could significantly decrease the impact of traumatic injuries of the CNS across a spectrum of severities and in both the immature and the adult brain. Notwithstanding the remaining questions highlighted above, whose answers will help optimise the interventions, the pragmatic approach that has already led to the first interventional studies in mild TBI confirms the promise of these compounds and shows the path ahead towards a precision medicine-informed effective intervention.
